# Identification of *Euglena gracilis* β-1,3-glucan phosphorylase and establishment of a new glycoside hydrolase (GH) family GH149

**DOI:** 10.1074/jbc.RA117.000936

**Published:** 2018-01-09

**Authors:** Sakonwan Kuhaudomlarp, Nicola J. Patron, Bernard Henrissat, Martin Rejzek, Gerhard Saalbach, Robert A. Field

**Affiliations:** From the ‡ Department of Biological Chemistry, John Innes Centre, Norwich Research Park, Norwich NR4 7UH, United Kingdom,; the § Earlham Institute, Norwich Research Park, Norwich NR4 7UZ, United Kingdom,; ¶ Architecture et Fonction des Macromolécules Biologiques, Aix-Marseille University, 163 Avenue de Luminy, 13288 Marseille, France,; ‖ CNRS, UMR 7257, 163 Avenue de Luminy, 13288 Marseille, France, and; the ** Department of Biological Sciences, King Abdulaziz University, Jeddah 23218, Saudi Arabia

**Keywords:** phosphorylase, gram-negative bacteria, polysaccharide, glycoside hydrolase, glycobiology, phylogenetics, algae, molecular evolution, gene transfer, beta-1,3-glucan, Euglena gracilis, orthologs, polysaccharide utilization loci

## Abstract

Glycoside phosphorylases (EC 2.4.x.x) carry out the reversible phosphorolysis of glucan polymers, producing the corresponding sugar 1-phosphate and a shortened glycan chain. β-1,3-Glucan phosphorylase activities have been reported in the photosynthetic euglenozoan *Euglena gracilis*, but the cognate protein sequences have not been identified to date. Continuing our efforts to understand the glycobiology of *E. gracilis*, we identified a candidate phosphorylase sequence, designated EgP1, by proteomic analysis of an enriched cellular protein lysate. We expressed recombinant EgP1 in *Escherichia coli* and characterized it *in vitro* as a β-1,3-glucan phosphorylase. BLASTP identified several hundred EgP1 orthologs, most of which were from Gram-negative bacteria and had 37–91% sequence identity to EgP1. We heterologously expressed a bacterial metagenomic sequence, Pro_7066 in *E. coli* and confirmed it as a β-1,3-glucan phosphorylase, albeit with kinetics parameters distinct from those of EgP1. EgP1, Pro_7066, and their orthologs are classified as a new glycoside hydrolase (GH) family, designated GH149. Comparisons between GH94, EgP1, and Pro_7066 sequences revealed conservation of key amino acids required for the phosphorylase activity, suggesting a phosphorylase mechanism that is conserved between GH94 and GH149. We found bacterial *GH149* genes in gene clusters containing sugar transporter and several other GH family genes, suggesting that bacterial GH149 proteins have roles in the degradation of complex carbohydrates. The Bacteroidetes *GH149* genes located to previously identified polysaccharide utilization loci, implicated in the degradation of complex carbohydrates. In summary, we have identified a eukaryotic and a bacterial β-1,3-glucan phosphorylase and uncovered a new family of phosphorylases that we name GH149.

## Introduction

Glycoside phosphorylases (EC 2.4.x.x) carry out the phosphorolysis of sugar polymers to produce the corresponding sugar 1-phosphates and shortened glycan polymer chains. However, the reaction is freely reversible *in vitro*, enabling the production of oligosaccharides from a simple sugar 1-phosphate and suitable carbohydrate acceptors with strict regio-, stereo-, and chain length specificity (1, 2). Despite the potential for use of the phosphorylases for oligosaccharide production, a relatively small number of these enzymes have been identified, limiting their range of applications. Nevertheless, the number of enzyme accessions is gradually increasing due to the substantial increase in available genome sequences and research efforts into characterization of novel phosphorylase activities.

Linear β-1,3-d-glucan polysaccharides are found across the prokaryotes and eukaryotes (3). The most well-known examples are bacterial curdlan (4), plant callose, and microalgal (*Euglena*) paramylon, all of which are high-molecular weight and water-insoluble. Paramylon, curdlan, and related compounds have been used in a wide range of applications: as part of anti-tumor and anti-HIV treatments (5, 6), as immune stimulants (7), and as alternative materials to fossil fuel products (8). Whereas the water insolubility of paramylon enables it to accumulate to up to 90% of *Euglena* cell mass (9), it creates complications for subsequent chemical modification, such as sulfation (10). Therefore, research into the generation of linear, soluble β-1,3-d-glucan polymers *in vitro* is of interest. Enzymatic transglycosylation using a mutant β-1,3-d-endoglucanase (11) or a partially purified β-1,3-d-glucan phosphorylase preparation from *Euglena gracilis* have been used for β-1,3-d-glucan production (12). The latter approach can be coupled with sucrose phosphorylase to generate β-1,3-d-glucan disaccharide, laminaribiose, from sucrose (13). Enzymatic synthesis of β-1,3-d-glucan using *Euglena* phosphorylases is attractive because of the relatively inexpensive substrates required (glucose and glucose 1-phosphate). However, the generation of the phosphorylase catalyst is time-consuming and laborious because it requires a large volume of *Euglena* cell culture that takes several days to grow. Moreover, the partial purification of the phosphorylase may result in loss of protein yield and attenuation of the activity.

**Figure 1.**
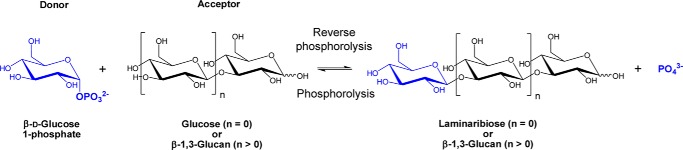
**Reaction carried out by β-1,3-d-glucan phosphorylase.**

Continuing our efforts to explore the glycobiology of *E. gracilis* (14–18) and our investigation into glycoside phosphorylases and their applications in enzymatic synthesis (19, 20), we focused on the established capability of the organism for β-1,3-d-glucan biochemistry, in particular its known β-1,3-d-glucan phosphorylase activities (EC 2.4.1.31) (Fig. 1). The enzymes have been categorized into two subgroups, based on the chain length specificity for their acceptor substrates: laminaribiose (β-1,3-glucobiose) phosphorylases (LBPs),^2^ and laminarin (laminaridextrin) or β-1,3-d-glucan phosphorylases (LDPs). LBP was previously considered to catalyze only a reversible phosphorolysis of laminaribiose (21), whereas LDPs have strong substrate preferences for β-1,3-d-glucans with DP 3 or greater (22). LBP activities were originally discovered in *E. gracilis* (23, 24) and *Astasia ocellata* (25) and subsequently in bacteria such as *Paenibacillus* sp. strain YM-1 (26) and *Acholeplasma laidlawii* (27). In contrast, LDP activities have only been described in the Euglenozoan *E. gracilis* (28) and in the heterokonts *Ochromonas malhemensis* (29) and *Ochromonas danica* (30). Despite the various reported β-1,3-d-glucan phosphorylase activities, LBP sequences are the only β-1,3-d-glucan phosphorylases that have been successfully cloned to date; they have been classified as members of glycoside hydrolase family GH94 in the CAZy database (www.cazy.org)^3^ (31). However, the eukaryote phosphorylases have not, to date, been classified due to the lack of sequence information, which compromises further biochemical and structural studies of this fascinating group of enzymes.

The physiological role of eukaryotic β-1,3-d-glucan phosphorylases remains inconclusive. In contrast, the biological function of the bacterial phosphorylases can be predicted from the identity of other genes with which they are clustered within the genome. LBP from *Paenibacillus* sp. strain YM-1 was hypothesized to be involved in degradation of laminaribiose, based on the position of the LBP genetic locus next to ABC sugar transporter components (26). Glycoside phosphorylase genes have also been observed in gene clusters containing other sugar transporters. For instance, a sucrose phosphorylase gene in human gut *Bifidobacterium lactis*, a sucrose transporter, and a transcriptional regulator form a sucrose-utilization gene cluster, which is transcriptionally up-regulated upon induction by sucrose and raffinose (32). A β-1,4-d-mannosyl-*N*-acetyl-d-glucosamine phosphorylase was reported as part of a gene cluster involved in *N*-glycan metabolism in *Bacteroides thetaiotaomicron* VPI-5482 and was shown to be involved in the phosphorolysis of β-1,4-d-mannosyl-*N*-acetyl-d-glucosamine into α-d-mannose 1-phosphate and *N*-acetyl-d-glucosamine (33). Two GH130 β-1,2-mannoside phosphorylases (Teth514_1788 and Teth514_1789) from *Thermoanaerobacter* sp. X-514 showed different chain-length specificities on phosphorolysis of β-1,2-oligomannan. The genes encoding Teth514_1788 and Teth514_1789 are located in a predicted GDP-d-mannose biosynthetic gene cluster. This finding demonstrates a possible role of Teth514_1788 and Teth514_1789 in a novel biosynthetic pathway, in contrast to the predicted degradative roles in other phosphorylases (34).

In the present work, a new family of phosphorylases was uncovered, designated GH149. Two GH149 sequences, a eukaryotic *E. gracilis* phosphorylase 1 (EgP1) and a bacterial metagenome sequence (Pro_7066), have been characterized and confirmed as β-1,3-d-glucan phosphorylases. GH149 contains several hundred sequences, most of which belong to Gram-negative marine bacteria from the phyla Proteobacteria and Bacteroidetes. The Bacteroidetes *GH149* genes map to previously predicted polysaccharide utilization loci (PULs) (35, 36), strongly suggesting a role for these GH149 enzymes in polysaccharide degradation by marine bacteria. Possible evolutionary relationships between the bacterial GH149 and the eukaryote GH149 are discussed based on phylogenetic analysis of the GH149 amino acid sequences and GC content analysis of the *GH149* genes.

## Results

### Identification of Euglena phosphorylase candidates through proteomic analysis

**Figure 2.**
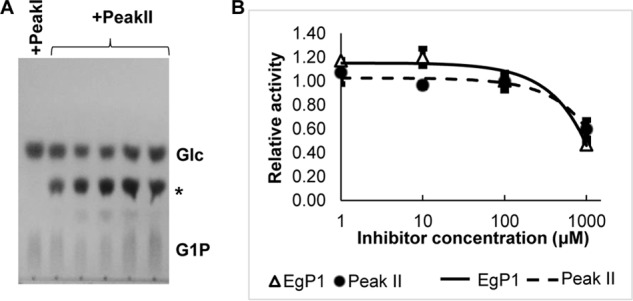
**Partial purification of *Euglena* phosphorylase from the cell-free extract.** *A*, TLC analysis of *Euglena* phosphorylase-mediated reaction between Glc and G1P. A disaccharide product was detected (*asterisk*). *B*, relative reverse phosphorolysis activity carried out by partially purified phosphorylase (Peak II) using Glc and G1P as substrates (*dotted line*) or recombinant EgP1 (*solid line*) in the presence of DNJ.

It has previously been hypothesized that *Euglena* β-1,3-glucan phosphorylase sequences may belong to GH94 family based on an activity somewhat similar to the GH94 bacterial laminaribiose phosphorylases (26, 27). To test this hypothesis, we used 20 characterized GH94 amino acid sequences as queries (Table S1) to interrogate the translated *Euglena* transcriptome (14) using tBLASTn (threshold = 0.0001). No sequences were recovered, suggesting that the *Euglena* phosphorylases may not be closely related to the members of the GH94 family as previously hypothesized. This is supported by previous comprehensive carbohydrate active enzyme (CAZyme) analyses on *Euglena* transcriptomes (14, 37), which did not identify any GH94 candidates. To identify the *Euglena* proteins with β-1,3-glucan phosphorylase activity, we performed a fractionation of *Euglena* protein extracts followed by proteomic analysis of the fractions with active phosphorylase activity. Cell-free extracts of 7-day-old, dark-grown *E. gracilis* were fractionated through anion-exchange chromatography (AIEX) as the first step of partial purification of β-1,3-glucan phosphorylase, based on previous publications (21, 24). The AIEX fractions were screened for phosphorylase activity in a reverse phosphorolysis reaction (Fig. 1) using a phosphate release assay with glucose (Glc) and α-glucose 1-phosphate (G1P) as an acceptor and a donor, respectively. The activity of the phosphorylase was detected in two sets of fractions, eluted at 18% (w/v) NaCl (Peak I) and 24–34% (w/v) NaCl (Peak II) (Fig. S1*A*). The oligosaccharide products of the reverse phosphorolysis reactions were analyzed by TLC, which showed production of a disaccharide only from the Peak II catalyzed reactions (Fig. 2*A*). The phosphorylase activity in Peak II could use laminaribiose (G2) and laminaritriose (G3) as acceptors (Fig. S1*B*), forming higher-DP oligosaccharides, which would suggest that this fraction contained an LDP activity.

Iminosugars, such as 1-deoxynojirimycin (DNJ), show weak competitive inhibition (*K_i_*/*K*′*_i_* = 570 μm, where *K_i_* and *K*′*_i_* represent the inhibitions against the free enzyme and the enzyme-substrate complex, respectively) against the phosphorylase activity of a GH94 cellobiose phosphorylase from *Cellvibrio gilvus* (38). To investigate whether there is an inhibition of the *Euglena* phosphorylase(s) by DNJ, the partially purified enzyme activity in Peak II was incubated with Glc and G1P in the presence of DNJ. DNJ showed weak inhibition in the 100–1000 μm range in the reverse phosphorolysis reaction (Fig. 2*B* and Fig. S1*B*), in keeping with the inhibition reported for the *C. gilvus* cellobiose phosphorylase.

Immobilized glycomimetic molecules, such as *N-*butyldeoxynojirimycin, have been used in affinity-enrichment proteomics to identify iminosugar-interacting proteins from mammalian tissue (39). Affinity-enrichment proteomics was performed using *N-*5-carboxypentyl-DNJ immobilized on agarose beads as a glycomimetic probe to identity DNJ-interacting phosphorylase candidates in the *Euglena* Peak II sample. The sample was applied to the *N*-5-carboxypentyl-DNJ matrix, followed by washing with buffer to remove unbound proteins. The binding proteins were eluted with DNJ-containing buffer. The eluted protein fractions were subjected to proteomic LC-MS/MS analysis. From a total of 253 proteins with unique peptide counts >3 and 100% protein identification probability, 240 were identified in the translated transcriptome database of *E. gracilis* (14) using Mascot (Matrix Science, Boston MA). Putative functions of these proteins were assigned by comparison with protein sequences with annotated functions in the non-redundant database NCBI by BLASTP pairwise alignment (File S1). One protein (m. 14570_dark) showed similarity to a deposited sequence, which was annotated by the depositor as a laminaridextrin phosphorylase-like protein from *E. gracilis* (BAV19384.1, *E*-value = 0, percentage identity = 53). The predicted molecular mass of m. 14570_dark is 130 kDa, which is similar to what has been reported for the partially purified *Euglena* laminaribiose phosphorylase (24). The translated *Euglena* transcriptome was interrogated using the m. 14570_dark sequence (designated *E. gracilis* phosphorylase 1; EgP1) as a BLASTP query, and three further sequences were identified with >70% sequence identity to m.14570_dark (m. 14571_dark, 14576_dark, and 14578_dark). These three candidate phosphorylases were designated EgP2, EgP3, and EgP4, respectively.

### Biochemical characterization of EgP1

To confirm the enzymatic activity of the EgP1–4, the recombinant proteins were produced for biochemical characterization. It was anticipated that heterologous expression of *Euglena* CAZymes might not be straightforward, given the problems encountered by the Suzuki group when trying to achieve productive expression of *Euglena* β-1,3-glucanase (40). An *E. coli* protein expression system was chosen for its simplicity and relatively short protein production time. Coding sequences were amplified from synthetic cDNA of EgP1–4, individually ligated into the Popin F plasmid vector (41). The recombinant plasmids containing EgP1–4 cDNA were then transformed into *E. coli* (Lemmo (DE3)), and expression was induced using 0.2 mm isopropyl 1-thio-β-d-galactopyranoside. Only expression of EgP1 was detectable. Initially, EgP1 formed a completely insoluble aggregate, which was purified by washing with buffer containing 6 m urea (Fig. S2*A*). The washed insoluble EgP1 suspension (Fig. S2*B*) unexpectedly showed phosphorylase activity when assayed with Glc and G1P in the reverse phosphorolysis reaction (Fig. S2*C*).

**Figure 3.**
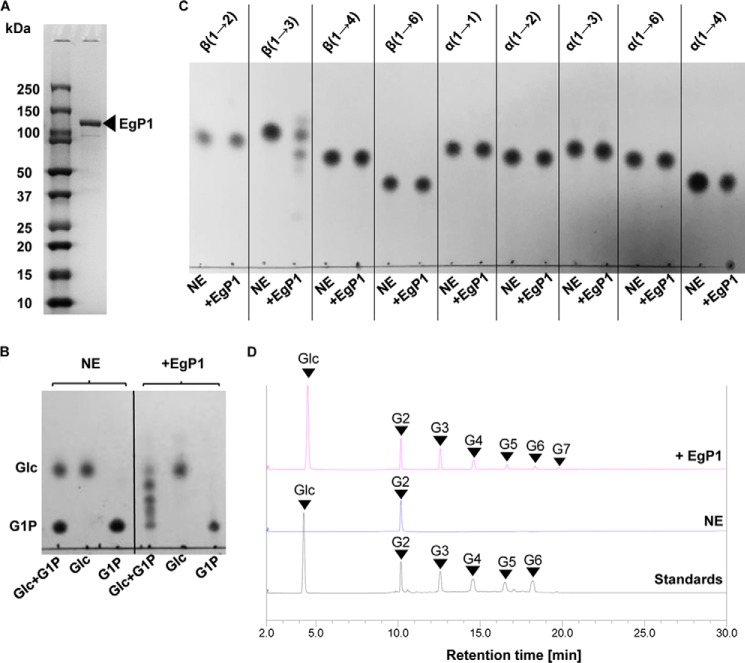
**Characterization of EgP1.** *A*, SDS-PAGE analysis of purified recombinant EgP1 after immobilized metal affinity chromatography and gel filtration. *B*, TLC analysis of the reverse phosphorolysis reaction catalyzed by EgP1. *C*, TLC analysis of the phosphorolysis reactions with glucose disaccharides linked by various glycosidic bonds. The linkages of the substrates are indicated at the *top. D*, HPAEC-PAD analysis of the phosphorolysis of G2 (Glc-β-1,3-Glc) to confirm the breakdown to Glc. However, due to the reversibility of the reaction, longer oligosaccharides (G3–G7) were detected. *NE*, no enzyme control.

Analysis of the EgP1 amino acid sequence using TargetP version 1.1 (http://www.cbs.dtu.dk/services/TargetP/)^3^ predicted the presence of an N-terminal mitochondrial targeting peptide at residues 1–31 (reliability class = 2), which we hypothesized could be the cause of protein aggregation. A truncated version of the EgP1 coding sequence was recloned without the predicted target peptide but with an N-terminal His_6_ tag, and the recombinant protein was expressed as described previously. A soluble EgP1 recombinant protein was detected, which was purified by immobilized metal affinity chromatography and gel filtration (Fig. 3*A* and Fig. S3) with a final yield of ∼30 mg/liter of *E. coli* culture.

The soluble EgP1 protein was able to catalyze the conversion of Glc and G1P into oligosaccharides with DP up to 12 glucose units that could be detected by MALDI-TOF after 1 h (Fig. S4 and Table S3). The EgP1 activity was dependent on both Glc and G1P as substrates; excluding either from the reaction mixture completely abolished oligosaccharide production (Fig. 3*B*). The recombinant EgP1 showed similar catalytic capability over a range of pH (pH 5–8) and a range of temperature (30–40 °C) (Fig. S5).

To investigate whether EgP1 activity is specific to β-1,3-linked glucan, phosphorolysis assays (Fig. 1) were performed against a variety of glucose disaccharides with various glycosidic linkages and anomeric configurations. Analysis of these reactions by TLC and high-performance anion-exchange chromatography with pulsed amperometric detection (HPAEC-PAD) showed that EgP1 was active exclusively on the Glc-β-1,3-Glc oligosaccharides, thus confirming its function as a β-1,3-d-glucan phosphorylase (Fig. 3, *C* and *D*). Kinetic data of the reverse phosphorolysis with Glc, G2, G3, laminaritetraose (G4), laminaripentaose (G5), or laminarihexaose (G6) as acceptors and G1P as a donor indicated that the enzyme preferred Glc and G2 as acceptors with similar catalytic efficiency (*k*_cat_*/K_m_* = 1.99 and 1.62 for Glc and G2, respectively). There was a nearly 2-fold reduction in *k*_cat_*/K_m_* as the chain length increased from G2 to G3, which is reflected in a 2-fold increase in the *K_m_* values (0.67 for G2 and 1.26 for G3). The catalytic efficiency continued to decrease with increasing chain length from G4 to G6, accompanied by an increase in the *K_m_* values for the respective acceptors (Table 1).

**Table 1:** **Kinetics parameters of EgP1 for the reverse phosphorolysis using G1P as a donor**

Acceptors	*k*_cat_	*K_m_*	*k*_cat_*/K_m_*
	*s*^−*1*^	*mm*	*s*^−*1*^ *mm*^−*1*^
Glc	1.10 ± 0.03	0.56 ± 0.06	1.99
G2	1.08 ± 0.03	0.67 ± 0.08	1.62
G3	1.12 ± 0.02	1.26 ± 0.09	0.89
G4	1.12 ± 0.03	1.41 ± 0.13	0.79
G5	1.13 ± 0.03	2.29 ± 0.19	0.50
G6	1.10 ± 0.03	2.88 ± 0.23	0.38

### Identification and characterization of a bacterial ortholog of EgP1

**Figure 4.**
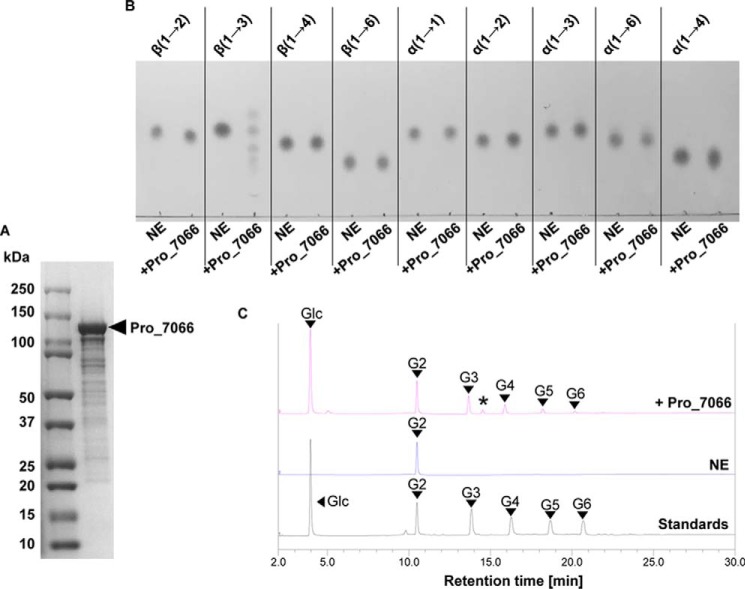
**Characterization of Pro_7066.** *A*, SDS-PAGE analysis of Pro_7066 after gel filtration. *B*, TLC analysis of the phosphorolysis reactions with glucose disaccharides with different linkages. The linkages of the substrates are indicated at the *top. C*, HPAEC-PAD analysis of G2 (Glc-β-1,3-Glc) phosphorolysis to confirm the breakdown to glucose. However, due to the reversibility of the reaction, the formation of G3–G6 oligosaccharides was also detected. *, small trace of G1P due to incomplete desalting by mixed-bed ion-exchange resin. *NE*, no enzyme control.

A proprietary metagenome database was interrogated with tBLASTN using EgP1 as a query.^4^ The search identified a putative ortholog, designated Pro_7066, with 45% sequence identity to EgP1 (*E*-value = 0). To investigate whether Pro_7066 has activity similar to that of EgP1, a recombinant form of Pro_7066 was produced in *E. coli* in the same manner as EgP1 (Fig. 4*A* and Fig. S6). Characterization of Pro_7066 was performed as described previously for EgP1, which showed that the enzyme was also active as a β-1,3-d-glucan phosphorylase (Fig. 4 (*B* and *C*) and Fig. S7 (*A* and *B*)), generating oligosaccharide detectable by MALDI-TOF up to DP 11 (Fig. S7*C* and Table S3). Pro_7066 showed similar pH and temperature preferences to EgP1 (Fig. S8). Interestingly, the kinetic parameters for the reverse phosphorolysis reaction with Glc-G6 as acceptors and G1P as a donor showed similar catalytic efficiency for all acceptors investigated, which is strikingly different from that observed in EgP1 (Table 2).

**Table 2:** **Kinetic parameters of Pro_7066 for the reverse phosphorolysis reaction using G1P as a donor**

Acceptors	*k*_cat_	*K_m_*	*k*_cat_*/K_m_*
	*s*^−*1*^	*mm*	*s*^−*1*^ *mm*^−*1*^
Glc	1.66 ± 0.04	0.29 ± 0.03	5.79
G2	1.54 ± 0.01	0.25 ± 0.02	6.03
G3	1.53 ± 0.02	0.37 ± 0.03	4.16
G4	1.39 ± 0.01	0.36 ± 0.02	3.89
G5	1.27 ± 0.01	0.32 ± 0.02	4.04
G6	1.18 ± 0.04	0.26 ± 0.04	4.62

### Multiple-sequence alignment and phylogeny of EgP1 and Pro_7066

The non-redundant protein sequence database (https://www.ncbi.nlm.nih.gov/protein/), the Microbial Eukaryote Transcriptome Sequencing Project (MMET) (7.8 × 10^6^ sequences) and the *E. gracilis* strain Z transcriptome (http://euglenadb.org/)^3^ have been interrogated with PSI-BLASTP and tBLASTN (threshold 0.0001) using the EgP1 amino acid sequence as a query (File S2). Several hundred sequences of bacterial origin with >30% sequence identity to EgP1 were identified in the non-redundant protein sequence NCBI database. Protein sequences of eukaryotic origin with 50–62% sequence identity to EgP1 (93–97% query coverage, *E*-value = 0) were identified in the MMET database and *E. gracilis* Z strain transcriptome.

**Figure 5.**
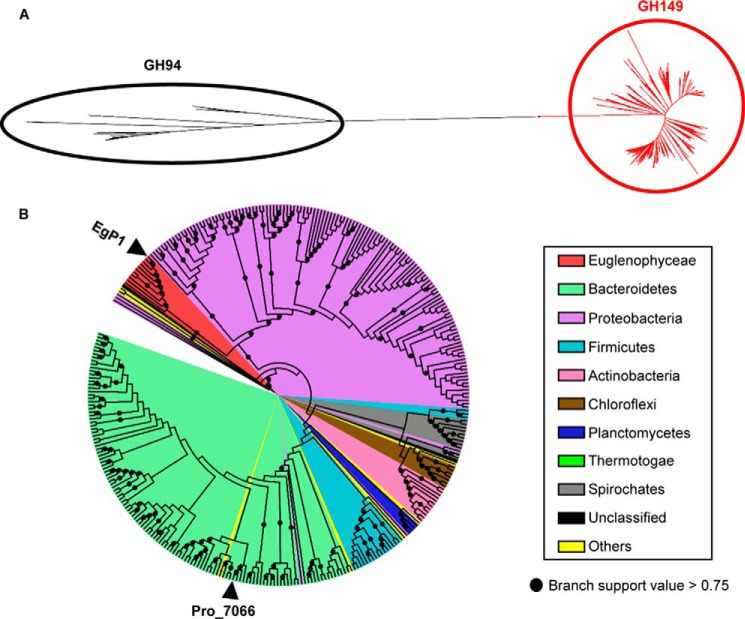
**Phylogenetic analysis of GH149.** *A*, an unrooted tree representing the phylogenetic relationship between GH94 queries (*black branches*) and GH149 (*red branches*). *B*, phylogenetic analysis of GH149. The *label colors* represent the taxonomic phyla of the species containing GH149. The positions of EgP1 and Pro_7066 are indicated by *arrowheads*.

**Figure 6.**
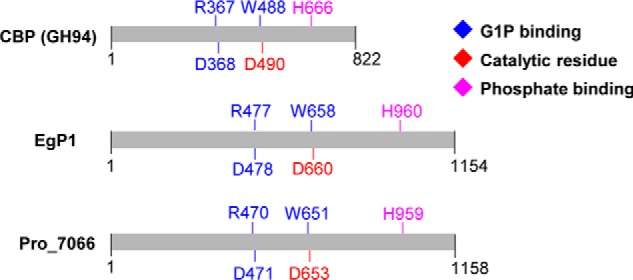
**Conserved amino acids required for phosphorylase activity.** The EgP1 sequence represents the truncated version where the predicted target peptide (residues 1–31) has been removed. *CBP*, cellobiose phosphorylase from *C. gilvus* (BAA28631.1).

A reconstructed molecular phylogeny showed that these EgP1 orthologous sequences formed a discrete clade (Fig. 5*A*, *red branches*), separate from GH94 sequences (Fig. 5*A*, *black branches*), thus forming a new glycoside hydrolase family, denominated GH149. To generate hypotheses about the active site residues of GH149, we aligned the amino acid sequences of EgP1–4 and Pro_7066 with those of members of family GH94. Interestingly, the amino acids that constitute the active site of GH94, including the −1 subsites (G1P binding), Asp catalytic residues, and residues for phosphate recognition (Fig. 6, Table S2, and Fig. S9), appeared conserved in GH149, suggesting that GH149 and GH94 form a clan that we term GH-Q.

Within the GH149 clade, all sequences from Euglenophyceae share a common node (Fig. 5*B*, *red label*, branch support value = 1) comprising EgP1–4, two sequences from *E. gracilis* strain Z (EuglenaDB IDs 750 and 1135, with 50 and 53% sequence identity to EgP1, respectively; *E*-value = 0), the laminaridextrin phosphorylase-like protein from *E. gracilis* strain NIES-47, and three sequences from *Eutreptiella gymnastica* CCMP1594 identified from MMET (CCMP1594 IDs 200380720, 200416338, and 200418968 with 62, 50, and 51% sequence identity to EgP1, respectively; *E*-value = 0).

Most bacterial GH149 family members were identified from sequenced and translated genomes of the phylum Proteobacteria, dominated by class γ-proteobacteria (Fig. 5*B*, 134 sequences, *purple labels*), and the phylum Bacteroidetes (Fig. 5*B*, 125 sequences, *green labels*). The majority of these sequences are currently annotated as predicted hypothetical proteins with unknown function. Pro_7066 was found to be most closely related to sequences from Bacteroidetes species (Fig. 5*B*, *black arrowhead* with *Pro*_*7066 label*), in particular *Flavobacteria bacterium* GWF1-32-7 (OGS61795.1, percentage identity = 76, *E*-value = 0), suggesting that Pro_7066 might belong to a *Flavobacteria* species. The Bacteroidetes species from which GH149 were found were predominantly isolated from marine environments; 13 have been described to be associated with algae and phytoplankton.

## Discussion

β-1,3-d-Glucan phosphorylase activities have previously been detected in both bacterial LBPs and eukaryote microalgal LDPs. However, only bacterial LBPs were fully characterized; their sequences were identified and categorized into the GH94 family. The lack of sequence information for eukaryotic β-1,3-d-glucan phosphorylases prevented heterologous expression and further characterization. It also prevented enzyme classification based on CAZy criteria and prohibited investigation of their relationship to other GH families. Identification of genetic sequences encoding proteins with β-1,3-d-glucan phosphorylase activity is therefore a crucial step in this study. DNJ affinity enrichment of *Euglena* protein extract with active β-1,3-d-glucan phosphorylase activity coupled with proteomic analysis was employed to identify a sequence candidate, designated EgP1. The EgP1 sequence was then used to interrogate a propriety metagenome database, identifying a second bacterial β-1,3-d-glucan phosphorylase candidate, designated Pro_7066. Both proteins were successfully expressed as recombinant proteins in *E. coli*, which allowed us to characterize their functions. Although more closely related to GH94 than to other GH families, EgP1 and Pro_7066 have no significant sequence identity to any characterized GH94 sequences, which suggests that they belong to a new CAZyme family, namely GH149.

*In vitro* characterization confirmed the function of EgP1 and Pro_7066 as β-1,3-d-glucan phosphorylases. EgP1 and Pro_7066 used Glc and longer β-1,3-linked gluco-oligosaccharides as acceptors in the reverse phosphorolysis reaction. This substrate specificity is slightly different from the previously described β-1,3-d-glucan phosphorylases, where substrate chain length was used to distinguish between different groups of the phosphorylases (*i.e.* LBPs and LDPs). The preference of EgP1 for Glc resembles the activity reported for LBP from semipurified *E. gracilis* extracts (23, 24). However, our findings are in agreement with more recent works by Ogawa *et al.* (12) and Muller *et al.* (13), which described a more relaxed chain length specificity of *Euglena* LBP. Therefore, the chain length specificity of β-1,3-glucan phosphorylases might be more flexible than previously observed. This has also been observed in a recently characterized thermophilic cellodextrin phosphorylase (β-1,4-glucan phosphorylase), which uses Glc and longer cello-oligosaccharide as acceptors (42) and is distinct from GH94 cellodextrin phosphorylases, which have no detectable activity on Glc as an acceptor (19, 43).

Comparisons of the kinetic parameters of EgP1 and Pro_7066 showed distinct preferences for the chain length of sugar acceptors; EgP1 preferred Glc and G2, and the *k*_cat_*/K_m_* values declined as the chain length rose to G6. Increasing acceptor chain length led to an increase in *K_m_* values, whereas *k*_cat_ values remained comparable. This result indicates that substrate binding (*K_m_*) dictates the acceptor chain length preference in EgP1 rather than the turnover (*k*_cat_) of the enzyme-substrate complex to product. In contrast, Pro_7066 showed comparable catalytic efficiency as well as *k*_cat_ and *K_m_* toward all acceptors. The contrasting kinetic parameters of EgP1 and Pro_7066 were surprising, considering that the two proteins are 45% identical. Structural studies of the two enzymes will help decipher the mechanistic details that may explain the difference in enzyme behaviors.

GH149 and GH94 members share conserved amino acids that are required for the phosphorylase activity but, overall, are not significantly similar. It is likely that phosphorylase activity in EgP1 and Pro_7066 sequences proceeds via a mechanism similar to that deciphered in GH94 enzymes (44–48). Therefore, GH149 and GH94 form a clan of related families, likely to have evolved from a common ancestor and diversified while retaining its key catalytic apparatus and substrate binding sites.

The GH149 family contains several hundred EgP1 orthologs from bacteria in the phyla Bacteroidetes and Proteobacteria and from eukaryotes in the phylum Euglenophyta, class Euglenophyceae. Phylogenetic analysis of GH149 sequences revealed a clade of Euglenophyceae sequences from *E. gracilis* and *E. gymnastica* under a common node. Phylogenetic analyses suggest a common origin for GH149 sequences found in *E. gracilis* (fresh water-living) and *E. gymnastica* (sea water-living), indicating that these sequences were inherited from a common Euglenophyceae ancestor before topographic isolation of *Euglena* and *Eutreptiella*. The origin of Euglenophyceae GH149 ancestral is unclear; however, the position of the Euglenophyceae clade within bacteria might suggest a lateral gene transfer from bacteria to the Euglenophyceae. A similar horizontal gene transfer from a bacterium to *E. gracilis* was predicted for mitochondrial *trans*-2-enoyl-CoA reductase (49). Nevertheless, it is important to note that bacteria are overrepresented in most available sequence databases compared with eukaryotes, and therefore the prevalence of the bacterial orthologs in our study is unsurprising. Increasing the availability of sequences from the diversity of unicellular eukaryotes is essential for investigating evolutionary origins.

A predicted targeting peptide in EgP1 caused aggregation of the EgP1 recombinant protein when expressed in *E. coli*. Removing this peptide from the sequence enabled solubility without affecting the expression level or activity of the protein. The targeting peptide length is consistent with the average length of predicted mitochondrial targeting peptides in *Euglena* previously reported by Krnáčová *et al.* (50), but it is still not known whether the targeting peptide is functional *in vivo*. Further investigation of the subcellular localization of the full-length EgP1 in *E. gracilis* cells is required to elucidate its biological function. In contrast, no target peptide was predicted for Pro_7066, and the length of this sequence was similar to that of the putative mature EgP1 protein (Fig. 6). Similarly, no targeting peptide was predicted for the GH149 sequences found in *Eutreptiella*.

**Figure 7.**
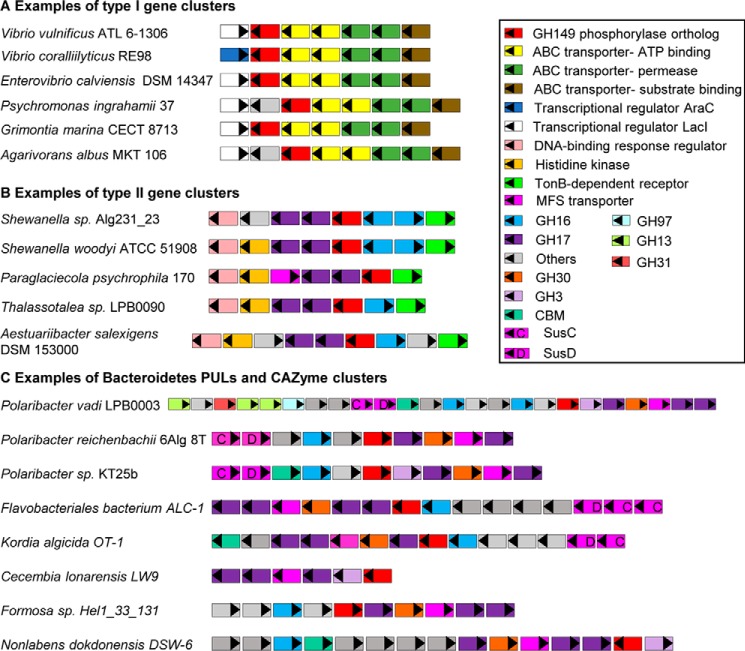
**Genetic organization of putative gene clusters containing the bacterial GH149 genes.** *A*, type I gene clusters contain ABC-transporter cassette, the phosphorylase, and a transcriptional regulator. *B*, Type II gene clusters contain a TonB-dependent receptor together with GHs and transcriptional regulators. *C*, predicted PULs and CAZyme clusters containing the putative phosphorylases from Bacteroidetes species.

Examination of the genomic location of *GH149* ORFs in Proteobacteria revealed their presence in putative gene clusters: Type I clusters containing ORFs for an ABC transporter cassette (a substrate-binding protein (COG0747), two permeases (COG0601 and 1173), and two ATP-binding proteins (COG0444 and 4608) and a transcriptional regulator LacI (COG1609) or AraC (COG2207)) (Fig. 7*A*). The same type of ABC transporter has been reported to be used by the thermophilic Gram-negative bacteria *Thermotoga maritima* for the utilization of extracellular laminarin after degradation by an extracellular laminarase. Oligosaccharide products from laminarase reactions are predicted to be taken up by the ABC transporter into the cytoplasm, where the oligosaccharides could be broken down into glucose by a laminaribiase (51). The lack of a gene encoding extracellular laminarase and an outer membrane transporter in Type I clusters suggests that this cluster may be involved in scavenging low-molecular weight β-1,3-gluco-oligosaccharides, such as laminaritriose/biose, using the putative phosphorylase as a catalyst. In our analysis, GH149 sequences were found in many *Vibrio* species, including *Vibrio campbellii* HY01, which has recently been reported to express a novel GH3 family β-glucosidase (LamN) capable of digesting laminaribiose (52). The co-occurrence of a GH149 and LamN in *V. campbellii* supports the utilization of β-1,3-glucan by *Vibrio* spp., reinforcing its importance in the carbon cycle of marine environments.

A second genetic co-localization pattern was also observed (designated Type II) mainly in genomes of bacteria in class γ-proteobacteria, order Alteromonadales. Type II clusters contain the respective *GH149* genes co-localized with a gene encoding a TonB-dependent receptor (cd01347), which is an outer membrane transporter involved in the transport of iron siderophore, vitamin B_12_, nickel complexes, and carbohydrates into the periplasmic space (53). Type II clusters also contain genetic components encoding other CAZymes (extracellular GH16 (pfam11721), cytoplasmic GH17 (COG5309)), regulators (histidine kinase (pfam06580) and DNA-binding response regulator (COG3279)), and an inner membrane major facilitator superfamily transporter (pfam13347) (Fig. 7*B*). A more complicated organization of the type II clusters suggests that they might be involved in degrading more complex β-1,3-glucan structures than that utilized by the type I cluster. Most importantly, the majority of *GH149* genes from Bacteroidetes map to the previously predicted PULs (clusters containing *susCD*-like gene pairs) and CAZyme clusters (clusters containing CAZymes but lacking *susCD*-like gene pairs) (Fig. 7*C*) (35, 54). The common features that are found in these PULs and CAZyme clusters are the presence of genes encoding β-glucosidases (GH3 and/or GH30_1) and glycanases able to hydrolyze β-1,3-glucans, GH17 and/or GH16, thus highlighting the potential contribution of the GH149 in the degradation of complex β-1,3-glucan structures.

The phylogenetic clustering of the bacterial GH149 amino acid sequences on Fig. 5*B* agreed with the taxonomic relationships of the species, suggesting that these bacterial *GH149* genes are probably inherited through a vertical gene transfer within each species. Comparison of the GC content of 52 bacterial *GH149* genes (File S3) showed limited deviation from the median GC content of the corresponding complete genome sequences, supporting the vertical gene transfer hypothesis.

In summary, the genetic loci containing *GH149* revealed co-localization of *GH149* with genes encoding several families of glycoside hydrolase as well as sugar transporters, suggesting the biological roles of GH149 protein in polysaccharide degradation, in particular β-1,3-glucan from external sources. Similar GC content of the *GH149* genes and its associated bacterial genomes suggests that the *GH149* genes were inherited through vertical gene transfer. Variation in the architectures of gene clusters containing the *GH149* genes suggests that the inherited *GH149* genes were independently associated into the gene clusters and PULs of individual species.

Our work supports the involvement of novel phosphorylases in β-1,3-glucan degradation and emphasizes the importance of gene clusters containing CAZymes and their roles in carbohydrate metabolism in marine Gram-negative bacteria. Our discovery of the eukaryote GH149 sequences would enable further determination of their physiological function in Euglenophyceae using genetic manipulation, which would subsequently aid our understanding of the involvement of the GH149 phosphorylase in β-1,3-glucan metabolism.

## Experimental procedures

### Euglena culture

*E. gracilis* was grown in 2 liters of EG:JM medium (replacing peptone and tryptone with casein hydrolysate (Sigma-Aldrich)) supplemented with 1% glucose for 7 days at 30 °C with gentle agitation in the dark.

### AIEX purification

Approximately 20 g wet weight of 7-day-old *Euglena* cells was harvested by centrifugation (500 × *g*, 5 min). The cell pellet was washed twice with MilliQ water and resuspended in a lysis buffer (50 ml, 20 mm HEPES, pH 7.0, 1 mg of ribonuclease A (Sigma), 1 tablet complete protease inhibitor mixture (Roche Applied Science)). The cells were lysed on ice by sonication, followed by centrifugation (32,914 × *g*, 30 min) to remove the cell debris. The supernatant was collected and filtered through a 0.2-μm disc filter (Millipore). A 5-ml AIEX column (Hitrap Sepharose Q, GE Healthcare) was pre-equilibrated with AIEX buffer (25 mm Tris-HCl, pH 8.5). The supernatant containing *Euglena* proteins (5 ml) was mixed with an equal volume of the AIEX buffer and loaded onto the pre-equilibrated AIEX column. The proteins were eluted with an NaCl gradient (0–700 mm) in the AIEX buffer over 20 column volumes and collected as 2-ml fractions.

### Enzyme assays

For the *Euglena* lysate or AIEX fractions, a phosphate release assay (55) was carried out in 20 μl of an assay buffer (100 mm HEPES, pH 7.0, 20 mm G1P, 10 mm acceptor (Glc, laminaribiose (G2), laminaritriose (G3)) and 200 mm sodium molybdate). The *Euglena* clear lysate or AIEX fractions (10 μl) were incubated with the assay buffer for 2 h at 30 °C. The reaction was stopped by boiling (5 min) and left to cool to room temperature. A color solution (90 μl, 0.1 m HCl, 13.6 m sodium ascorbate) was added to the boiled reaction mixture and incubated for 30 min at room temperature to allow color development. A stop solution (90 μl, 68 mm sodium citrate tribasic dihydrate, 2% acetic acid) was added to the mixture to stop the color development. The absorbance of final solution was measured at 620 nm on a 96-well plate reader. The amount of phosphate release was calculated from the absorbance by comparing with a phosphate standard curve ranging between 0 and 10 mm. All assays were performed in triplicates. Kinetic parameters of EgP1 and Pro_7066 were determined using the phosphate release assay (20 μl) with the enzymes (25 μg/ml) in the presence of 0.2–10 mm Glc G2, G3, G4, G5, or G6 and 10 mm G1P. The amount of phosphate release from the assays was measured, and the values were fitted on non-linear regression with a Michaelis-Menten model using GraphPad Prism to determine *V*_max_ and *K_m_*.

A phosphorolysis assay was carried out using 20 μl of 20 mm oligosaccharides, 10 mm KH_2_PO_4_ in 20 mm HEPES, pH 7.0, and 10 μl of AIEX fractions, which were desalted to remove any endogenous phosphate by passing through a PD-10 column (GE Healthcare) or with 1 μl of EgP1 or Pro_7066 (10 mg/ml stock solution). The reaction mixture was incubated for 1 h at 30 °C. The reaction was stopped by boiling (5 min), and oligosaccharide products were analyzed by TLC analysis or HPAEC-PAD.

### DNJ affinity column preparation

The protocol was modified from Ref. 39; *N-*5-carboxypentyl-DNJ (50 μmol; Toronto Research Chemicals) was conjugated to resin (1 ml; Bio-Rad Affidex 102 resin) overnight in anhydrous methanol using 1-ethyl-3-(3-dimethylaminopropyl)carbodiimide (10 mg; Bio-Rad) as a catalyst. The reaction was performed at room temperature with gentle agitation. The conjugated resin was poured into a glass column and washed with 10 × 4 ml of water to remove methanol. The column was stored at 4 °C in aqueous 0.02% sodium azide solution before use.

### DNJ-affinity chromatography

The DNJ column was pre-equilibrated with 4 × 4 ml of water and 4 × 2 ml of washing buffer (20 mm HEPES, pH 7.0). Protein solution (800 μl) was loaded onto the column. Unbound proteins were washed with 2 ml of the washing buffer and 2 ml of 100 mm NaCl in the washing buffer. Bound proteins were eluted with an increasing concentration of DNJ (50 nm, 1 μm, 50 μm, and 1 mm) in the washing buffer (2 ml of each concentration) and collected as 2-ml fractions. The protein fractions were concentrated by freeze-drying. The concentrated fractions were pooled and analyzed by LC-MS/MS.

### Proteomic analysis

Proteins were purified from the supernatant using OMIX C4 tips (Agilent, Cheadle, UK). The amount of protein was determined using the Direct Detect® spectrometer (Merck Millipore, Watford, UK). Protein mixture (8 μg) was dissolved in 30 μl of 0.1 m TEAB buffer (Sigma-Aldrich) and 0.2% Rapigest (Waters, Manchester, UK), reduced with DTT, alkylated with iodoacetamide, and digested with 0.4 μg of sequencing grade trypsin (Promega, Southampton, UK) at 37 °C for 16 h. The reaction was stopped by adding TFA, and aliquots were analyzed by nano-LC-MS/MS on an Orbitrap Fusion^TM^ Tribrid^TM^ mass spectrometer coupled to an UltiMate® 3000 RSLCnano LC system (Thermo Scientific, Hemel Hempstead, UK). The mixtures were separated on a PepMap^TM^ 100 C18 LC column (C18, 2 μm, 500 × 0.75 mm; Thermo) using a gradient of 0.75% min^−1^ acetonitrile from 6 to 40% in water, 0.1% formic acid at a flow rate of 0.3 μl min^−1^ and infused directly into the mass spectrometer. The mass spectrometer was run in positive ion mode, with no quadruple isolation, at 120K resolution over the mass range 350–1800 (*m*/*z*) for the precursor scans (orbitrap). One microscan of 50 ms with an AGC target of 2*e*^5^ was used. MS2 threshold was set to 1.5*e*^4^, and precursors were fragmented by both collision-induced dissociation and higher-energy collisional dissociation with collision energy = 30 eV and an isolation window of 1.6 Da (quadrupole) using the automatic maximum speed option with ion injection for all available parallelizable time. Dynamic exclusion was set to 1 count and 30 s. Recalibrated peak lists were generated using MaxQuant version 1.5.2.8 (www.MaxQuant.org),^3^ and the database search was performed with the merged higher-energy collisional dissociation and collision-induced dissociation peak lists using Mascot version 2.4 (Matrixscience, London, UK). The search was performed on a *E. gracilis* protein database (14) with a precursor tolerance of 6 ppm and a fragment tolerance of 0.6 Da. The enzyme was set to trypsin/P with a maximum of 2 allowed missed cleavages. Carbamidomethyl (C) was set as a fixed modification, and oxidation (M) and acetylation (protein N terminus) were used as variable modifications. The Mascot search results were imported into Scaffold version 4.4.1.1 (www.proteomesoftware.com)^3^ using identification probabilities of 99 and 95% for proteins and peptides.

### Bioinformatics analyses

Orthologous sequences to EgP1 were obtained from the non-redundant protein sequence database (https://www.ncbi.nlm.nih.gov/protein/), the Marine Microbial Eukaryote Transcriptome (MMET) Sequencing Project), and the *E. gracilis* Z strain transcriptome (http://euglenadb.org/) using BLASTP or tBLASTn with *E*-value score of 0.0001 or more. Multiple-sequence alignments of amino acid sequences were performed using Clustal Omega (56) (www.clustal.org/omega,^3^ version 1.2.2) with the default settings and edited with trimAl version 1.2 using a heuristic automated method (57). The alignments were visualized by Jalview (version 14.6.4) (58). Phylogenetic trees were reconstructed from a matrix of 331 unambiguously aligned amino acids from 325 species using PhyML version 3.0 (59) with the best fit model as inferred by a smart model selection. Bootstrap values were determined from a population of 100 replicates. Tree annotation and visualization were performed using iTOL version 3.4.3 (60). The GenBank^TM^ codes of the GH149 members can be found in File S4.

### Recombinant protein production

The *EgP1* cDNA sequence was synthesized and optimized for *E. coli* expression (custom DNA synthesis by Gen9, Inc.). The sequence was amplified by PCR and cloned into the PopinF plasmid vector (41) using In-Fusion^TM^ (TakaraBio, Mountain View, CA) following the manufacturer's protocol. The recombinant *PopinF-EgP1* was transformed into *E. coli* (Lemmo21), and a 1-liter culture of the transformant was grown at 25 °C in LB medium with agitation (180 rpm) overnight. Heterologous protein expression was induced by adding isopropyl 1-thio-β-d-galactopyranoside to a final concentration of 0.2 mm and incubated for 2 days at 18 °C. A recombinant plasmid pET28a containing *Pro*_*7066* cDNA (Prozomix Ltd.), Pro_7066, was transformed into *E. coli* (BL21 (DE3)) and grown as described previously for EgP1. The cells were harvested (6721 × *g*, 10 min) and lysed by sonication in buffer A (20 mm HEPES, pH 7.0, 250 mm NaCl) supplemented with 1 mg/ml DNase (Sigma) and 1 tablet of complete protease inhibitor mixture (Roche Applied Science). Supernatant containing the recombinant proteins was separated from cell debris by centrifugation (32,914 × *g*, 30 min). Proteins were purified with the ÄKTA Pure FPLC system (GE Healthcare) at 4 °C. The supernatant containing either His_6_-tagged EgP1 or Pro_7066 was loaded to a 1-ml HisTrap^TM^ HP column (GE Healthcare) pre-equilibrated with buffer A (10 mm HEPES, pH 7.5, 250 mm NaCl). The column was washed with buffer A, and bound proteins were eluted in one step with 10 mm HEPES, pH 7.5, 250 mm NaCl, 500 mm imidazole. The proteins were further purified by gel filtration using a Superdex S200 16/600 column (GE Healthcare) eluted with 20 mm HEPES, pH 7.5, 150 mm NaCl, 1 ml/min. Fractions containing the proteins were pooled and concentrated to 10 mg/ml using an Amicon Ultra-15 30,000 molecular weight cutoff concentrator. The proteins were stored in aliquots at −80 °C until required. The EgP1 nucleotide sequence was deposited to GenBank^TM^ with accession number MG516599.

### Oligosaccharide analyses

TLC was performed by spotting 0.5 μl of the recovered reaction mixture onto a silica plate (10 × 5 cm) and then elution using a mobile phase containing NH_4_OH/H_2_O/isopropyl alcohol (3:1:4) in a sealed glass container for 2 h to allow oligosaccharide separation. The plate was air-dried and stained with orcinol, which was prepared by adding concentrated sulfuric acid (20 ml) to an ice-cold solution of 3,5-dihydroxytoluene (360 mg) in ethanol (150 ml) and water (10 ml). The stained plate was then heated until oligosaccharide spots were visible.

HPAEC-PAD analyses were performed by diluting the reaction mixtures in MilliQ water to a final volume of 500 μl and desalted by mixed-bed ion-exchange resin (Sigma). The desalted mixtures were filtered through a disposable PTFE 0.45-μm filter disc (Merck Millipore) and subjected to HPAEC-PAD analysis using a Dionex ICS3000 chromatography system equipped with PAD and controlled by Chromeleon® software. A PA100 CarboPac column (analytical: 4 × 250 mm, guard: 4 × 50 mm) was used for all analyses. The solutions for elution of the oligosaccharides were as follows: solution A, 100 mm sodium hydroxide; solution B, 100 mm sodium hydroxide + 400 mm sodium acetate. The separation was achieved by gradient elution (0–100% solution B) from 1 to 30 min, followed by 100% B (30–50 min) and then re-equilibration of the column with solution A (50–60 min). The solutions were delivered to the column at a rate of 0.25 ml/min.

## Author contributions

S.K., N.J.P., and R.A.F. conceptualization; S.K., B.H., and G.S. data curation; S.K., N.J.P., M.R., and G.S. formal analysis; S.K. and G.S. investigation; S.K., M.R., G.S., and R.A.F. methodology; S.K. writing-original draft; S.K., N.J.P., B.H., M.R., G.S., and R.A.F. writing-review and editing; N.J.P. and R.A.F. supervision; B.H. and R.A.F. validation; R.A.F. funding acquisition; R.A.F. project administration.

## Supplementary Material

Supporting Information

## Footnotes

3 Please note that the JBC is not responsible for the long-term archiving and maintenance of this site or any other third party hosted site.

4 Prozomix Ltd., personal communication.

## Footnotes

2 The abbreviations used are:

LBP: laminaribiose (β-1,3-glucobiose) phosphorylase
LDP: laminarin (laminaridextrin) or β-1,3-d-glucan phosphorylase
DNJ: 1-deoxynojirimycin
Glc: glucose
G1P: α-glucose 1-phosphate
DP: degree of polymerization
PUL: polysaccharide utilization locus
CAZyme: carbohydrate active enzyme
AIEX: anion-exchange chromatography
HPAEC-PAD: high-performance anion-exchange chromatography with pulsed amperometric detection
G2: laminaribiose
G3: laminaritriose
G4: laminaritetraose
G5: laminaripentaose
G6: laminarihexaose.
