# Supplementary material for: Identification of Euglena gracilis β-1,3-glucan phosphorylase and establishment of a new glycoside hydrolase (GH) family GH149
Source: J Biol Chem. 2018 Jan 9;293(8):2865–76. doi: 10.1074/jbc.RA117.000936 (PMC5827456; doi:10.1074/jbc.RA117.000936)
Supplement: Supporting Information [file supp_293_8_2865__index.html]

Identification of Euglena gracilis β-1,3-glucan phosphorylase and establishment of a new glycoside hydrolase (GH) family GH149 — New β-1,3-glucan phosphorylase family — Identification of Euglena gracilis β-1,3-glucan phosphorylase and establishment of a new glycoside hydrolase (GH) family GH149 — New β-1,3-glucan phosphorylase family — Supporting Information 

# Identification of *Euglena gracilis* β-1,3-glucan phosphorylase and establishment of a new glycoside hydrolase (GH) family GH149

## Supporting Information

- Supplemental Data File 2 - BLAST analyses of GH149 amino acid sequences against EgP1
- Supplemental Data File 3 - GC content analysis of bacterial GH149 DNA sequences
- Supplemental Data File 4 - Sequence IDs for GH149 members
- Supplemental Data File 1 - List of Euglena proteins identified from affinity proteomics and their predicted functions
- Supplemental Data - Supplemental figures and tables
