# Supplementary material for: Identification of Euglena gracilis β-1,3-glucan phosphorylase and establishment of a new glycoside hydrolase (GH) family GH149
Source: J Biol Chem. 2018 Jan 9;293(8):2865–76. doi: 10.1074/jbc.RA117.000936 (PMC5827456; doi:10.1074/jbc.RA117.000936)
Supplement: Supporting Information [file supp_RA117.000936_133879_1_supp_40417_p1cx9m.pdf]

Supplemental Data

Identification of *Euglena gracilis* β-1,3-glucan phosphorylase and establishment of a new glycoside hydrolase (GH) family GH149

Sakonwan Kuhaudomlarp[a], Nicola J. Patron[b], Bernard Henrissat[c,d,e], Martin Rejzek[a], Gerhard Saalbach[a] & Robert A. Field[a,1]

[a]Department of Biological Chemistry, John Innes Centre, Norwich Research Park, Norwich NR4 7UH, UK. [b]Earlham Institute, Norwich Research Park, Norwich, NR4 7UZ, UK. [c]Aix-Marseille University, Architecture et Fonction des Macromolécules Biologiques, 163 Avenue de Luminy, 13288 Marseille, France. [d]Centre National de la Recherche Scientifique, UMR 7257, 163 Avenue de Luminy, 13288 Marseille, France. [e]Department of Biological Sciences, King Abdulaziz University, Jeddah, Saudi Arabia.

Running title: New β-1,3-glucan phosphorylase family

[1]To whom correspondence should be addressed: Robert A. Field: Department of Biological Chemistry, John Innes Centre, Norwich Research Park, Norwich NR4 7UH, UK. rob.field@jic.ac.uk.

Supplemental Table S1. Characterized GH94 sequences that were used for BLAST analysis with EgP1 and Pro_7066

**Sequence names***	**Genbank accession**
CBP from *Cellvibrio gilvus* ATCC 13127	BAA28631.1
ChBP from *Vibrio proteolyticus*	BAC87867.1
CBP from *Cellulomonas uda*	AAQ20920.1
CBP from *Ruminiclostridium thermocellum*	AAL67138.1
CBP from *Saccharophagus degradans*	ABD80168.1
CDP from *Ruminiclostridium thermocellum*	BAB71818.1
LBP from *Halorhabdus tiamatea.*	WP_020936056.1
CBP from *Clostridium stercorarium*	AAC45510.0
CDP from *Clostridium stercorarium*	AAC45511.1
CBP from *Cellvibrio gilvus* ATCC 13127	BAA28631.1
CBP from *Thermotoga maritima* MSB8	AAD36910.1
CBP from *Thermotoga neapolitana*	AAB95491.2
N,N'-diacetylchitobiose phosphorylase from *Vibrio furnissii*	AAG23740.1
ChBP from *Vibrio proteolyticus*	BAC87867.1
LBP from *Acholeplasma laidlawii* PG-8A	ABX81345.1
LBP from *Paenibacillus* sp. YM1	BAJ10826.1
N,N'-diacetylchitobiose phosphorylase from *Ruminococcus albus*	WP_013499018.1
CBP from *Halorhabdus tiamatea* SARL4B	CCQ33375.1
NdvB protein from *Neurospora crassa* OR74A	EAA28929.1
CellAP from *Xanthomonas campestris*	WP_011039146.1

*CBP = cellobiose phosphorylase, ChBP = chitobiose phosphorylase, CDP = cellodextrin phosphorylase, LBP = laminaribiose phosphorylase, CellAP = cellobionic acid phosphorylase

Supplemental Table S2. Summary of the observed $[M+Na]^+$ of glucans in MALDI-ToF analysis.

**DP of glucan**	**Calculated $[M+Na]^+$**	**The closest observed $[M+Na]^+$**
G4	689.221	689.203
G5	851.274	851.223
G6	1013.326	1013.287
G7	1175.379	1175.330
G8	1337.432	1337.374
G9	1499.485	1499.483
G10	1661.538	1661.517
G11	1824.581	1823.591
G12	1986.723	1985.649

Supplemental Table S3. Summary of conserved amino acid residues that are involved in GH94 catalysis and G1P binding that have been found in the EgP1 and Pro_7066

**GenBank accession**	**CAZy families**	**Catalytic residues**	**G1P binding (-1 subsite)**	**Phosphate recognition**	**References**	**PDB structures**
BAA28631.1	GH94	D490	W488, R367, D368	H666	(44)	2CQS, 2CQT
BAC87867.1	GH94	D492	W490, R349, D350	H644	(45)	1V7V, 1V7W, 1V7X
AAQ20920.1	GH94	D490	W488, R367, D368	H666	(46)	3RRS, 3RSY, 3S4A, 3S4B
AAL67138.1	GH94	D483	W481, R360, D361	H653	(47)	3QDE
ABD80168.1	GH94	D472	W470, R349, (N350)	H626	(48)	4ZLE, 4ZLF, 4ZLG, 4ZLI
BAB71818.1	GH94	D624	W622, R486	H817	(19)	5NZ7, 5NZ8
WP_020936056.1	GH94	D535	W533, R384, D385	H749	Inferred from Figure S9	NA
EgP1 (in this study)	GH149	D660	W658, R477, D478	H960	Inferred from Figure S9	NA
Pro_7066 (in this study)	GH149	D653	W651, R470, D471	H959	Inferred from Figure S9	NA

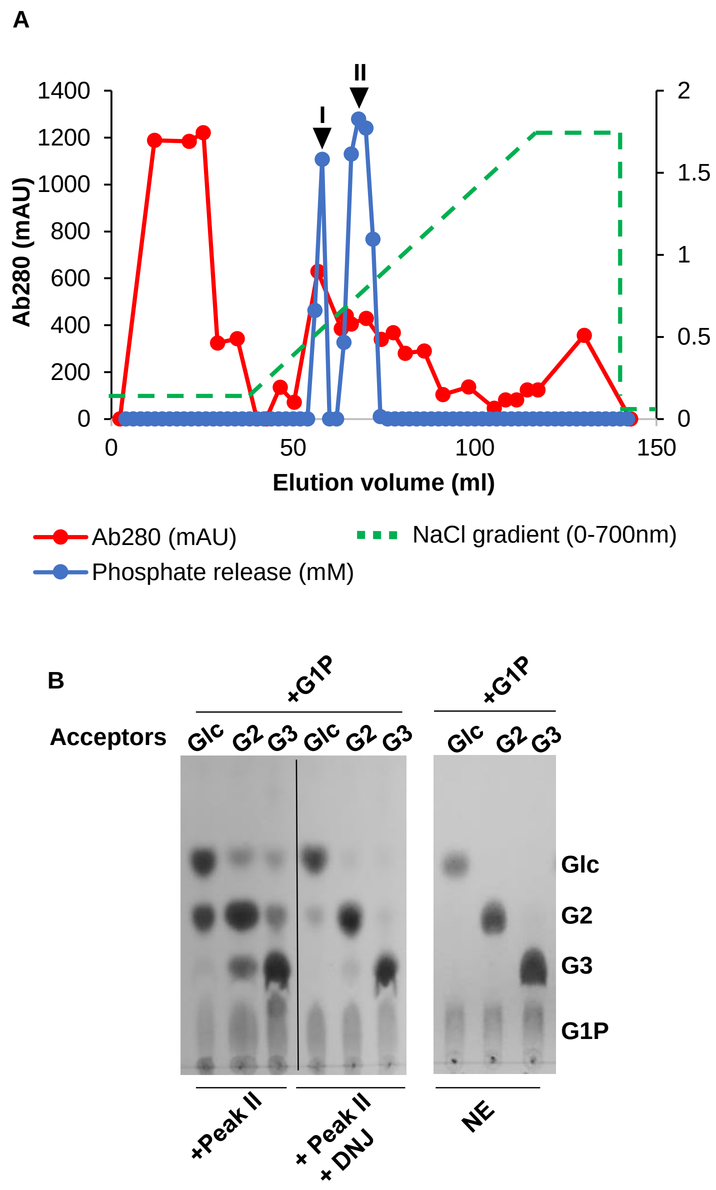

Fig. S1 Partial purification of Euglena phosphorylase from *E. gracilis* protein extract. (A) An overlay of AIEX elution profile (red) and the amount of phosphate release by the phosphorylase activity in each fraction in the phosphate release assay (blue). (B) TLC analysis of Euglena phosphorylase (PeakII) mediated reactions between G1P and laminaribiose (G2), or laminaritriose (G3) as substrates in the presence (+DNJ, 1000 µM) and absence of DNJ. NE = no enzyme control.

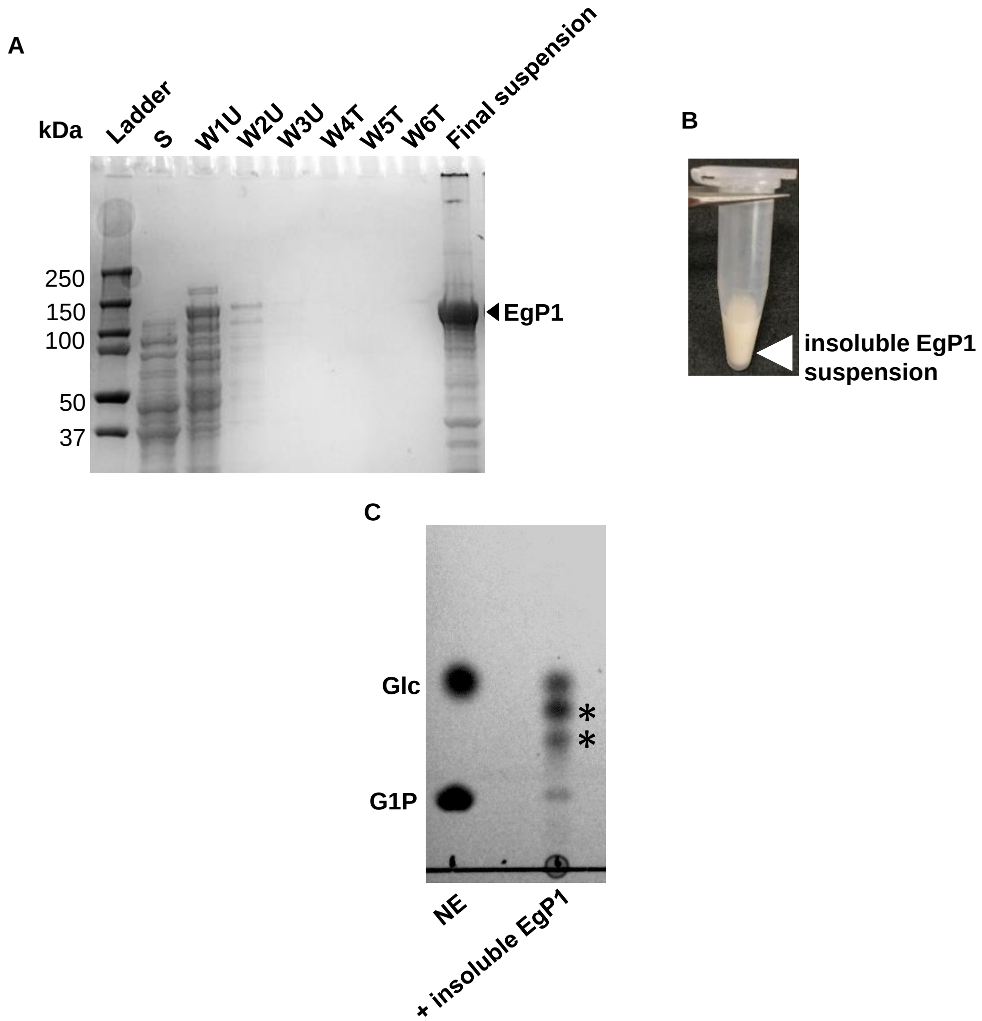

Fig. S2 Expression and characterization of insoluble recombinant EgP1. (A) SDS-PAGE analysis of insoluble EgP1after purification by washing with 50 mM Tris-HCl pH 7.5 containing 6 M urea (W1U-W3U) followed by washing with buffer alone (W4T-W6T, 50 mM Tris-HCl pH 7.5). (B) the final suspension of insoluble EgP1 in 50 mM Tris-HCl pH 7.5 buffer. (C) TLC analysis of the reverse phosphorolysis reaction carried out by the insoluble EgP1 with 10 mM Glc and 20 mM G1P. * = oligosaccharide products from the reverse phosphorolysis reaction.

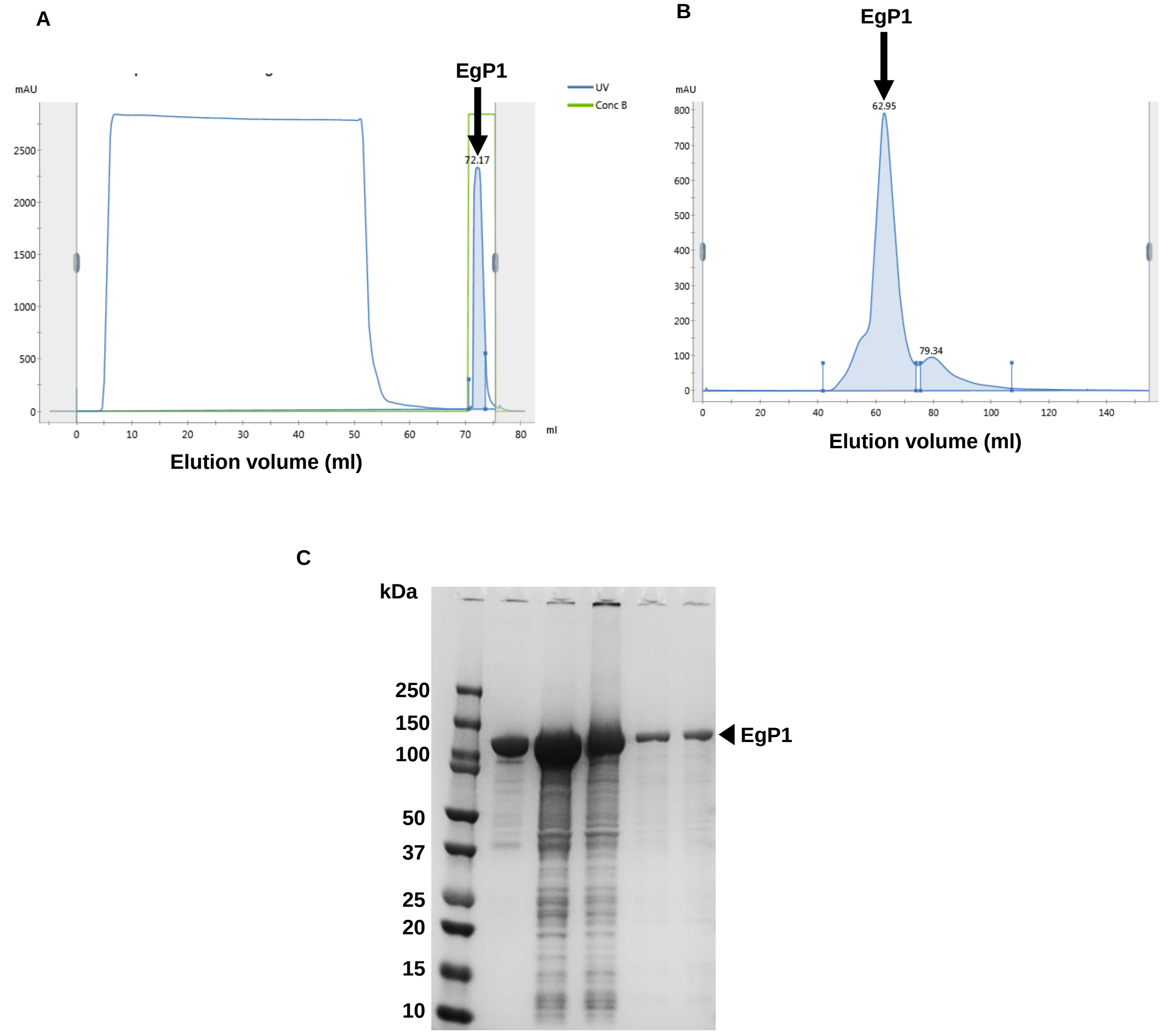

Fig. S3 IMAC and gel filtration purification of recombinant EgP1. (A) IMAC purification with UV detection (blue line). EgP1 was eluted in 100% of 10 mM HEPES pH 7.5, 250 mM NaCl, 500 mM imidazole (green line)). (B) Gel filtration of EgP1. The protein was eluted at 63 ml elution volume in 20 mM HEPES pH 7.5, 150 mM NaCl. (C) SDS-PAGE of EgP1 after IMAC purification.

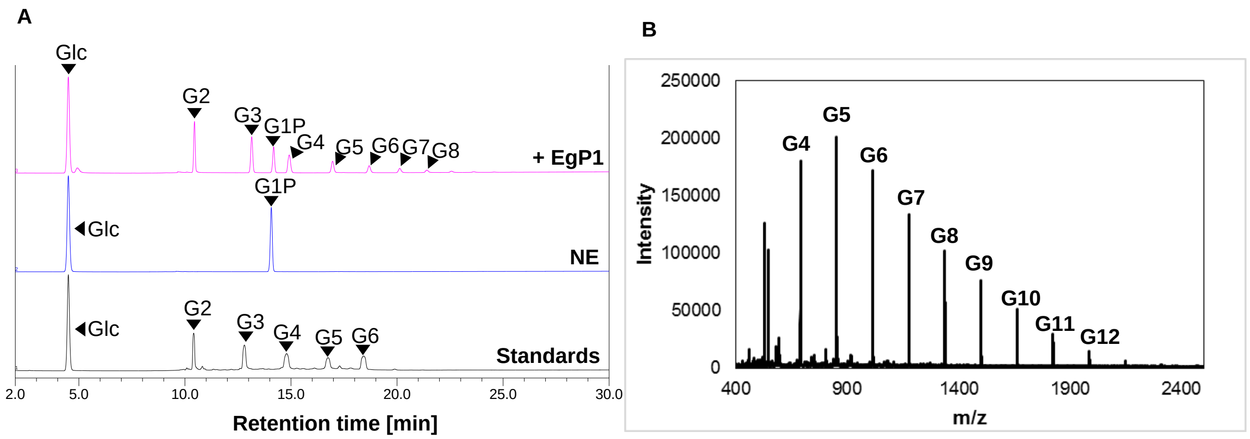

Fig. S4 Reverse phosphorolysis reaction catalyzed by EgP1. (A) HPAEC-PAD analysis of the reverse phosphorolysis carried out by EgP1 in the presence of Glc and G1P as substrates. (B) MALDI-ToF analysis after 1 hour.

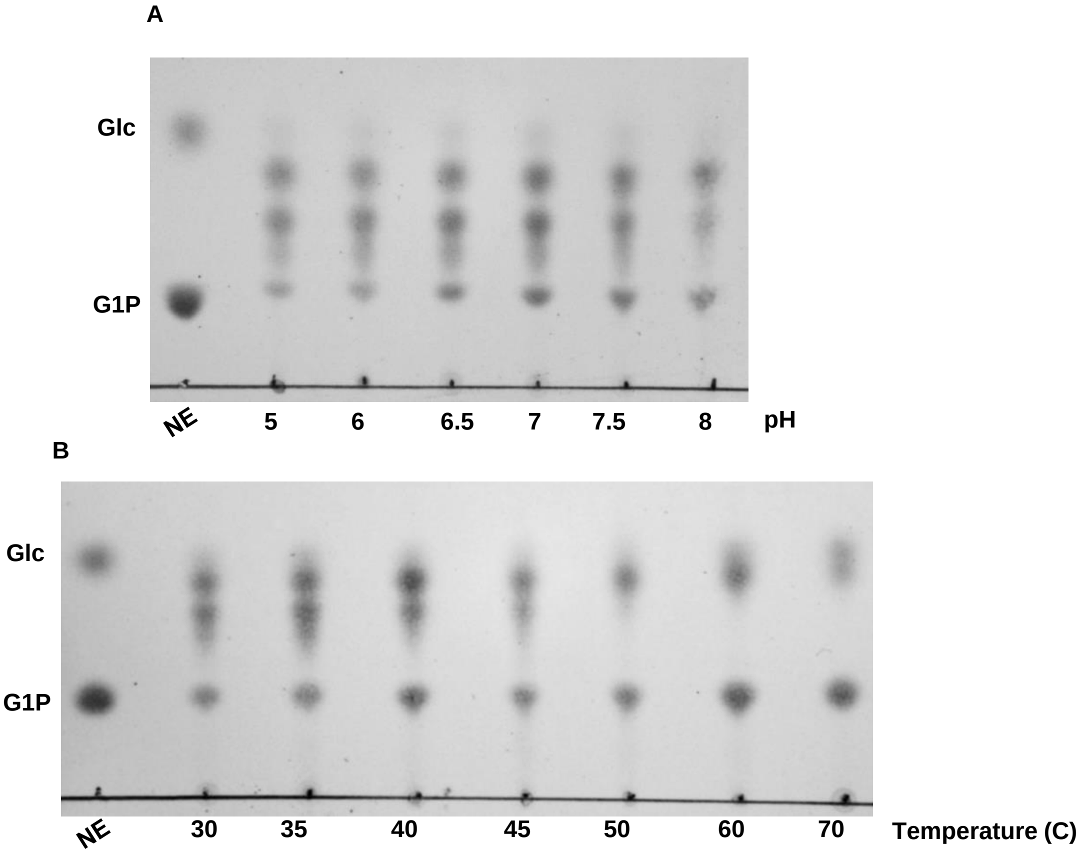

Fig. S5 TLC analysis of the reverse phosphorolysis carried out by EgP1 over a range of pH (A) at 30 °C or a range of temperatures (B) at pH 7.0.

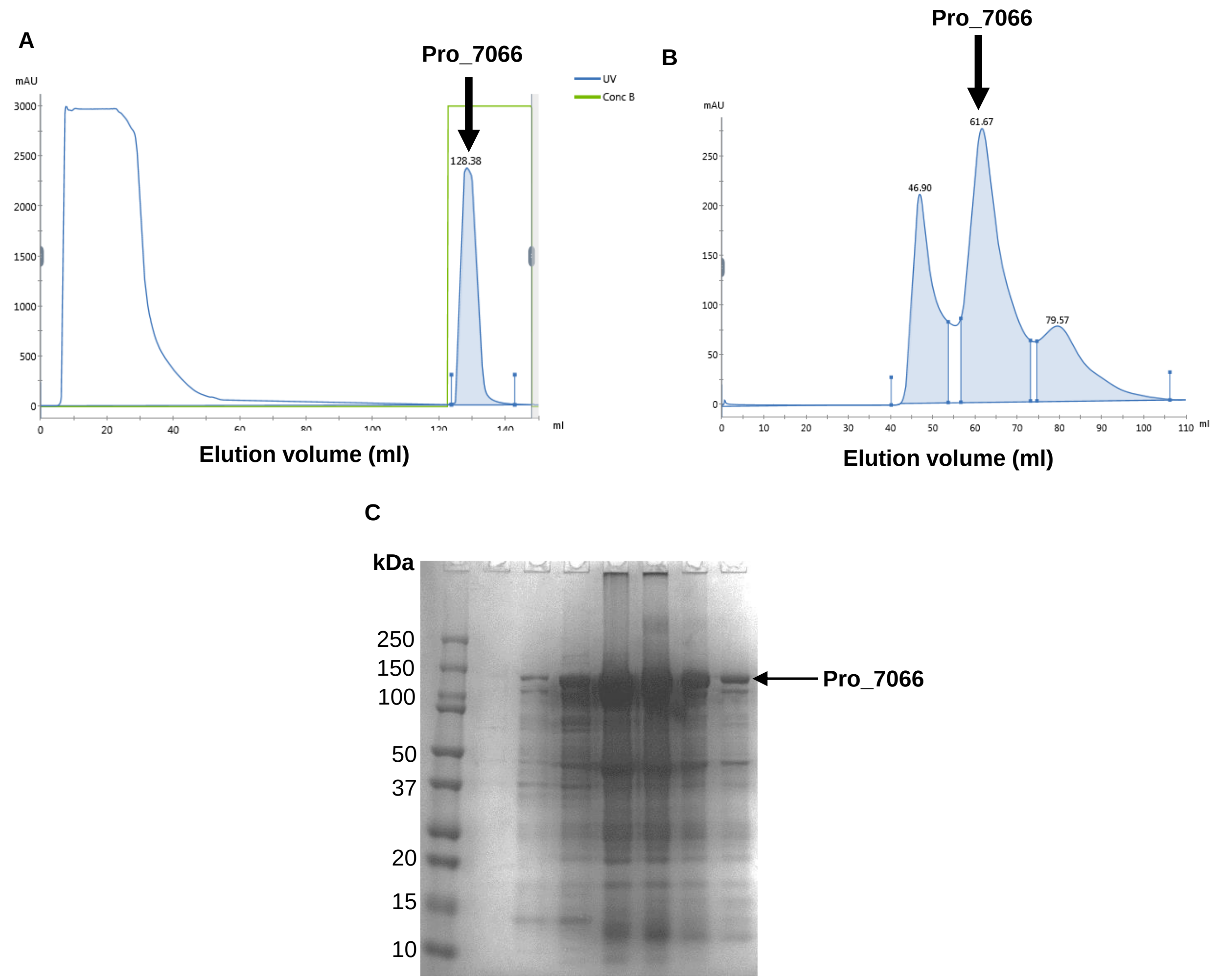

Fig. S6 IMAC and GF purification of recombinant Pro_7066. (A) IMAC purification with UV detection. Pro_7066 was eluted in 100% of 10 mM HEPES pH 7.5, 250 mM NaCl, 500 mM imidazole. (B) Gel filtration of Pro_7066. The protein was eluted at 63 ml elution volume in 20 mM HEPES pH 7.5, 150 mM NaCl. (C) SDS-PAGE of Pro_7066 after IMAC purification.

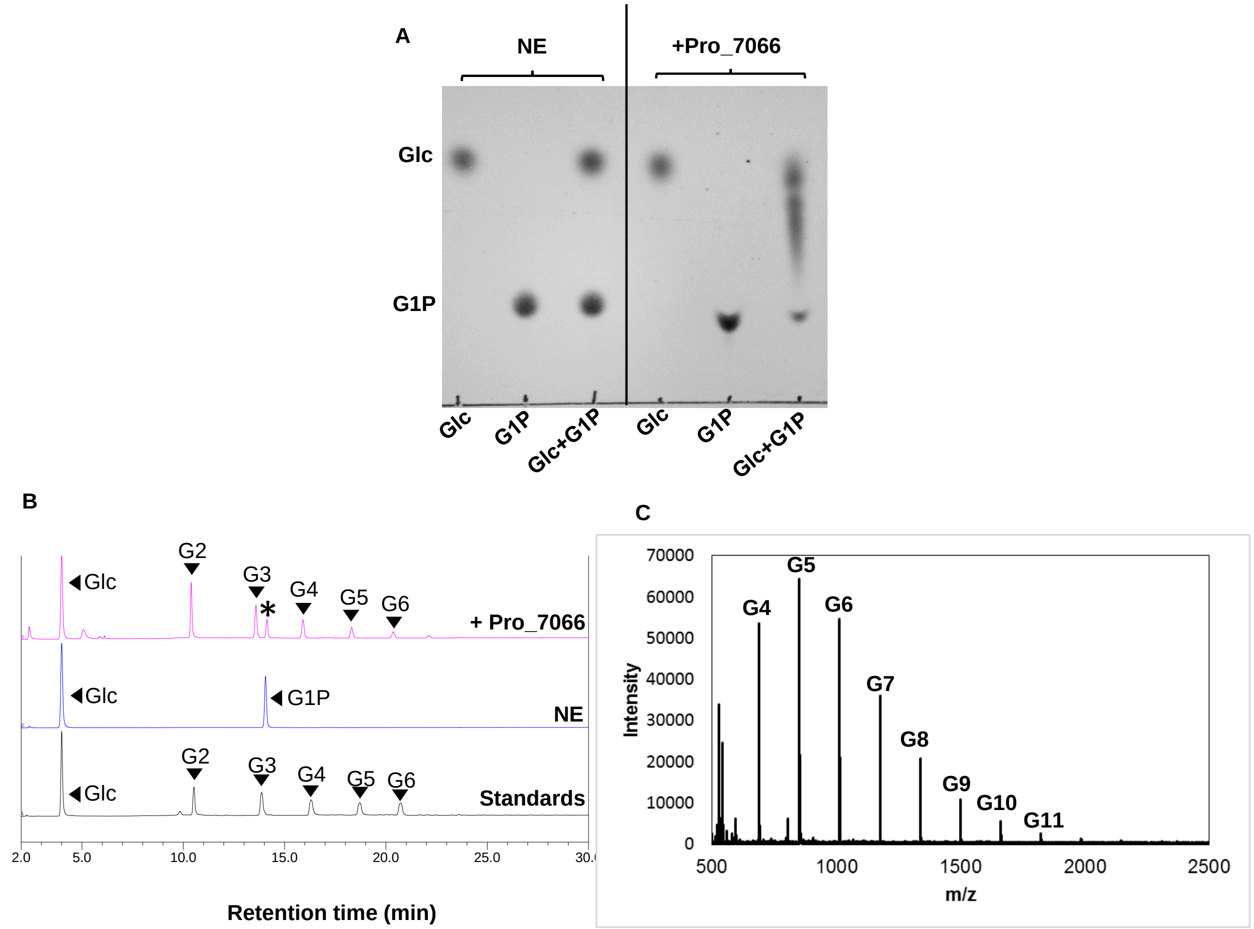

Fig. S7. Reverse phosphorolysis reaction catalyzed by Pro_7066 (A) TLC analysis of the reverse phosphorolysis carried out by Pro_7066. (B) HPAEC-PAD analysis of the reaction. (C) MALDI-ToF analysis of the reverse phosphorolysis after 1 hr. Asterisk = G1P.

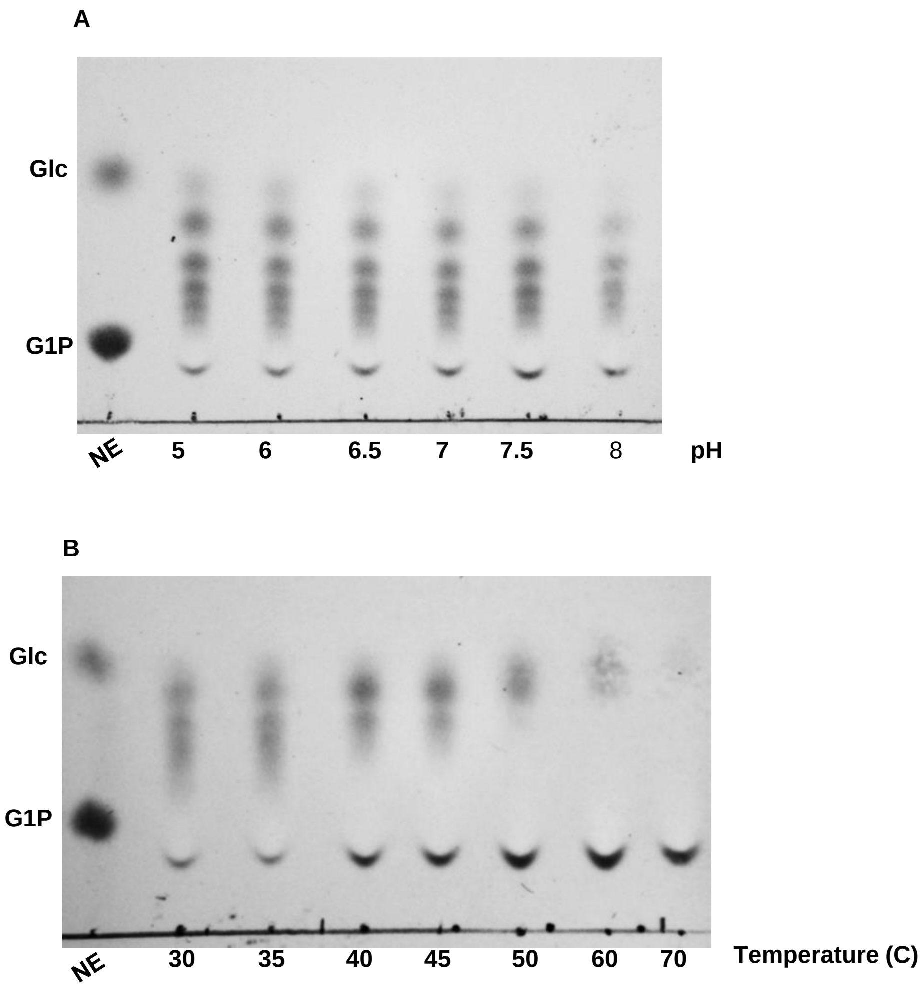

Fig. S8 TLC analysis of the reverse phosphorolysis carried out by Pro_7066 (A) over a range of pH at 30 °C or a range of temperatures (B) at pH 7.0.

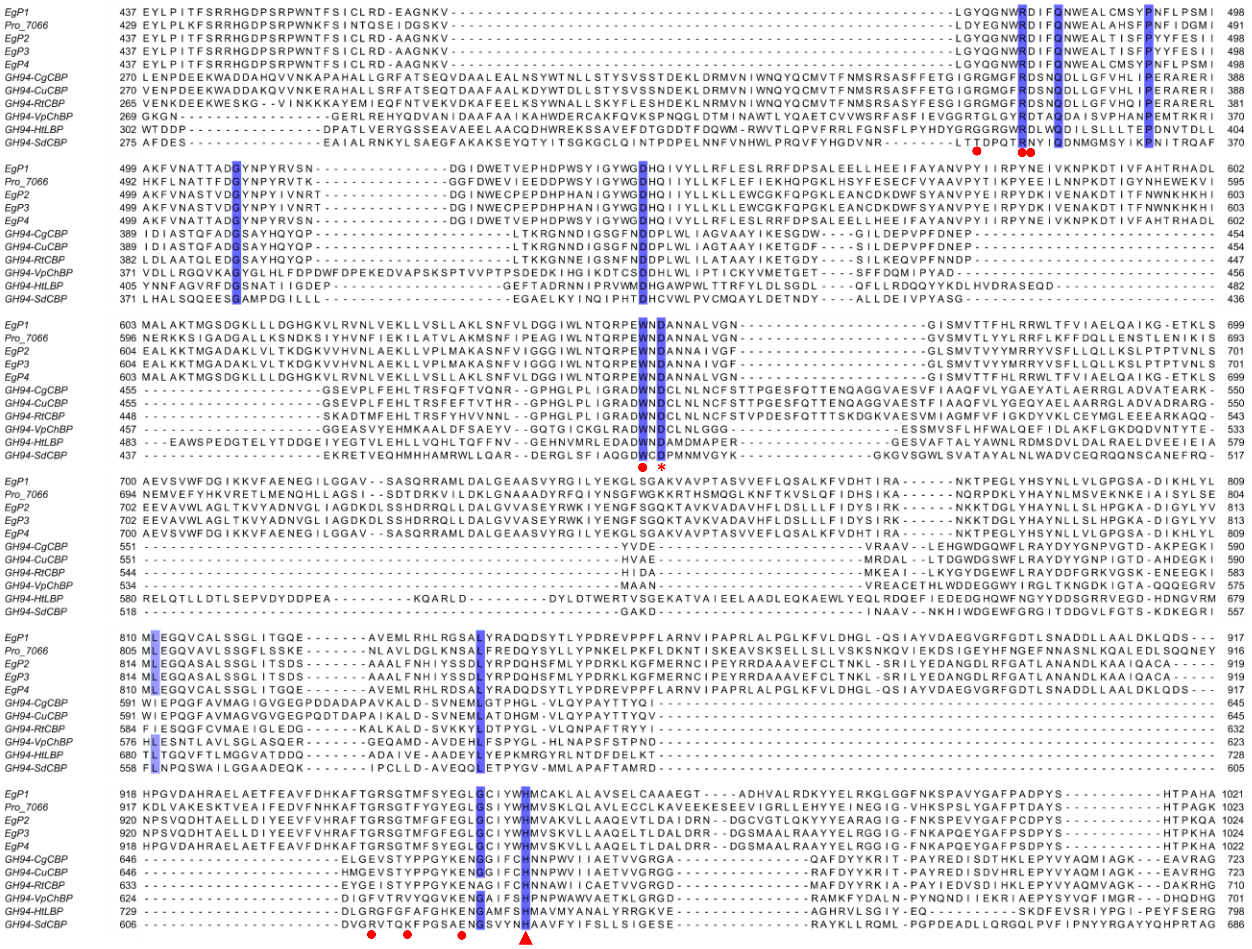

Fig. S9 Multiple alignment of EgP1-4, and Pro_7066 and GH94 enzymes revealed conservation of key amino acids required for the phosphorylase activity. Only a snap shot of the alignment is presented showing key amino acid residues. 100% conserved amino acid residues are highlighted in dark blue. Red circles indicated the residues involved in the subsite formation for sugar and sugar phosphate binding. Red triangle represents the residue involved in phosphate binding. Red asterisk represents the Asp catalytic residue.

List of supplemental data files

Supplemental Data File 1 – List of Euglena proteins identified from affinity proteomics and their predicted functions

Supplemental Data File 2 – BLAST analyses of GH149 amino acid sequences against EgP1

Supplemental Data File 3 – GC content analysis of bacterial GH149 DNA sequences

Supplemental Data File 4 – Sequence IDs for GH149 members
